# Traffic Accident Data Generation Based on Improved Generative Adversarial Networks

**DOI:** 10.3390/s21175767

**Published:** 2021-08-27

**Authors:** Zhijun Chen, Jingming Zhang, Yishi Zhang, Zihao Huang

**Affiliations:** 1 Intelligent Transportation Systems Research Center, Wuhan University of Technology, Wuhan 430070, China; chenzj556@whut.edu.cn (Z.C.); jeremy.zhang@whut.edu.cn (J.Z.); 2 School of Computer and Artificial Intelligence, Wuhan University of Technology, Wuhan 430070, China; 3 School of Management, Wuhan University of Technology, Wuhan 430070, China; yszhang@whut.edu.cn; 4 School of Civil Engineering and Transportation, South China University of Technology, Guangzhou 510640, China

**Keywords:** generative adversarial networks, traffic accident recognition, data characteristics, recognition accuracy

## Abstract

For urban traffic, traffic accidents are the most direct and serious risk to people’s lives, and rapid recognition and warning of traffic accidents is an important remedy to reduce their harmful effects. However, research scholars are often confronted with the problem of scarce and difficult-to-collect accident data resources for traffic accident scenarios. Therefore, in this paper, a traffic data generation model based on Generative Adversarial Networks (GAN) is developed. To make GAN applicable to non-graphical data, we improve the generator network structure of the model and used the generated model to resample the original data to obtain new traffic accident data. By constructing an adversarial neural network model, we generate a large number of data samples that are similar to the original traffic accident data. Results of the statistical test indicate that the generated samples are not significantly different from the original data. Furthermore, the experiments of traffic accident recognition with several representative classifiers demonstrate that the augmented data can effectively enhance the performance of accident recognition, with a maximum increase in accuracy of 3.05% and a maximum decrease in the false positive rate of 2.95%. Experimental results verify that the proposed method can provide reliable mass data support for the recognition of traffic accidents and road traffic safety.

## 1. Introduction

The transportation industry is growing rapidly, and road transport has been the most important mode of transportation today. However, a large number of road traffic accidents occur on the road every year, especially those caused by motor vehicles in highways and urban beltways, often leading to massive loss of life and property. According to WHO, road traffic accidents kill 1.35 million people worldwide each year, and road traffic is the leading cause of death among children and adolescents from the ages of 5 to 29. At present, due to the characteristics of urban traffic, such as high traffic flow and mixed traffic, once a traffic accident occurs it will seriously affect the efficiency of traffic flow, intensify urban congestion, and serious traffic accidents can even lead to paralysis of urban traffic. These will have a direct impact on the daily travel and lives of residents. If the scope of the accident increases, the direct result will be a huge loss of life and property. In this study, we only focus on vehicle accidents that occurred on highways and beltways where pedestrians or non-motors are forbidden.

At present, the traffic accident has become the main traffic problem affecting the safety of urban travel, while the rapid recognition and early warning of a traffic accident is one of the effective means to reduce accident losses. In past research of accident recognition, the lack of accident data was always a difficult problem to solve, and it was difficult for the traditional traffic accident recognition algorithm to establish a reliable abnormal recognition model through a large amount of data. The accuracy of the model algorithm still has much room for improvement, so the acquisition of traffic accident data has become an important means to improve the accuracy of traffic accident recognition. The traffic accident data have also become the major factor that affects the accuracy rate of traffic accident recognition.

The technique of generating a large number of similar samples from a small number of samples is called the data generation technique. This technique continuously trains the neural network model with deep learning methods. After the model learns the data distribution of the samples, it can resample to obtain more samples that meet the requirements to achieve the purpose of expanding the samples. After learning the probability distribution of data obtained from a large number of samples, the deep learning algorithm can automatically perform difficult tasks such as data classification [1], data repair [2], data mining [3,4], and data generation.

The best among these is Generative Adversarial Networks (GAN) [5], proposed by Ian Goodfellow in 2014, which generates images that mimic human handwritten numbers by training two neural networks that game each other. The images generated by using this technique can be exactly the same as the original images, generating new fonts that do not exist in the original samples and retaining the characteristics of the original samples. The technique is currently being developed and improved to achieve the goal of generating data for scarce medical samples [6], such as generating image data for cancer nodules, medical CT images, skin lesions, etc., and greatly improving the recognition of lesion samples with deep learning models.

The data augmentation technique based on deep learning utilizes the powerful computing ability of computers to effectively learn the distribution patterns of data. It solves the problems caused by insufficient sample size by generating completely new data samples. Inspired by this, we develop in this study a traffic accident data generation model based on Generative Adversarial Networks (GAN). Specifically, we improve the generator network structure of GAN to make it applicable to non-graphical data and use the generated model to resample the original data to obtain new traffic accident data. The proposed method is expected to provide reliable mass data [7] support for the recognition of traffic accidents and road traffic safety.

The remainder of this paper is organized as follows: Section 2 reviews the research on GAN and introduces other related approaches for data generation and augmentation. Section 3 proposes the improvement scheme of GAN for traffic accident scenarios. Section 4 introduces the experimental steps of accident data generation, the acquisition and preprocessing of the dataset, and the methods used in the comparative experiments. Section 5 analyzes the results of the experiments and shows the effectiveness of the generated data by the statistical test and the accident recognition task. Section 6 summarizes the research contributions, insights, and future research.

## 2. Literature Review

Data generation is a method of obtaining new samples by resampling the original data distribution. Among the existing high-performance data generation models, the Generative Adversarial Networks (GAN) proposed by Goodfellow is the main one, and several variants are derived to solve different data generation tasks. GAN can simulate the distribution of the real dataset and generate new data samples with high quality. Therefore, some extant work applies GAN as a data augmentation technique [8]. GAN is used by generators to learn the data distribution patterns of real samples in the data generation process [9]. Powerful data generation capabilities are obtained by fitting sample distribution functions by neural networks. A well-trained GAN can be directly applied to generate data samples that are consistent with the real data distribution, e.g., it can generate images, speech, video, and so on. Some studies show that GANs can also be used to solve deep learning problems when there is insufficient labeled data, such as generating labeled medical samples and labeled image data.

Subsequently, Mirza et al. propose Conditional GAN (CGAN), where additional information input into the generator model and discriminator model can convert GAN from unsupervised learning to supervised training [10,11]. Arjovsk et al. propose the Wasserstein GAN (W-GAN) and find that the gradient disappears during GAN training reasons, and theoretically prove the shortcomings of the original W-GAN using JS scatter to measure the distance between distributions and propose an optimization scheme [12]. Miyato et al. propose the Spectral Normalization GAN (SNGAN), which can solve the problem of unstable data generation from the restricted network level [13]. Martineau et al. propose the Relativistic GAN (RGAN) to illustrate that the probability that the real data are true should be reduced while improving the realism of the image data, and also that the training of RGAN is more stable [14]. Li et al. propose a histogram-based GAN (His-GAN), which helps GAN to generate high-quality generated data. The map generated data and original data into a histogram, then they modified the GAN training process by providing a set of samples to GAN one at a time, thus ultimately generating data that were more indistinguishable from the original data [15]. Qi et al. propose the Loss-sensitive GAN (LS-GAN) to solve the overfitting problem of the model by using s minimum quadratic loss function that limits the modeling ability [16]. The Large-Scale GAN (Big GAN) proposed by the Deep Mind team can increase the size of the network and the amount of training data, which becomes the best network in the current generation network [17]. Mohammad et al. propose a semi-recurrent hybrid VAE-GAN for generating sequential data that takes into account spatial correlation in the data, using CNN, and maintains the dependencies between frames, resulting in better audio data [18]. In the field of water transportation, Chen et al. improve the Generative Adversarial Networks to generate a large number of small boat image samples and used them for small boat recognition and showed the accuracy of the method through a large number of experiments [19,20,21,22]. Liu et al. propose a flexible data augmentation strategy by generating synthetically degraded images using CNN to enlarge the volume and diversity of the original dataset to train learning-based ship detection methods [23,24]. In the field of road traffic, although some scholars have studied the causes of bicycle, e-bikes [25,26], and pedestrian accidents [27] on urban roads to reduce the incidence of urban traffic accidents, a large amount of traffic accident data is still needed to predict traffic accidents as support. Wu et al. propose a scene generation model based on sequential Generative Adversarial Networks (LSTM-GAN), combining long short-term memory (LSTM) networks to capture the dynamics involved in temporal traffic flows. Spatio-temporal scenes of traffic flow that match the observed traffic flow characteristics of the road network can be well generated [28]. However, there is still a problem of insufficient data sample size. Solutions using Generative Adversarial Networks are still in their infancy, and research on the generation of raw traffic data has yet to be developed.

## 3. Materials and Methods

### 3.1. Generative Adversarial Networks

GAN is a neural network model with a novel structure proposed by Goodfellow et al. in 2014 [5], which is an excellent generative model for generating pictures similar to the original samples. In recent years, GAN has demonstrated amazing data generation effects, generating images with details, textures, and clarity to a level that cannot be distinguished by the human eye. Various network structure variants and theoretical approaches have been proposed, which have become the core research direction in the field of deep learning.

GAN introduces the concept of adversarial training in the basic framework. The model consists of two parts, the Generator and Discriminator network. The purpose of training the generator network is to model the distribution of the raw data and generate samples that are as close to the real data as possible.

**Figure 1:**
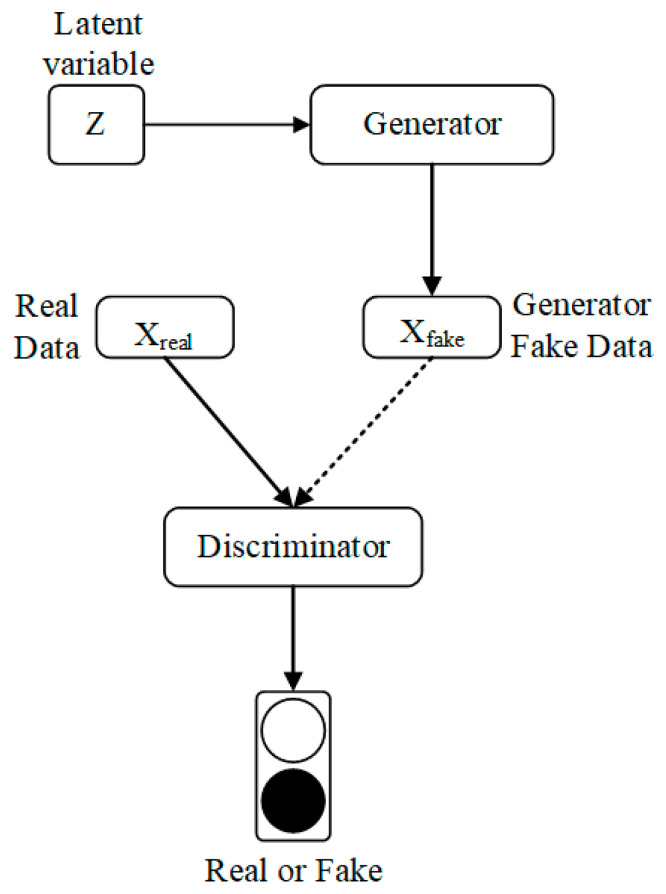
Schematic diagram of the structure of GAN.

The Generator network and discriminator network in the first proposed GAN consist of a fully connected neural network and the specific structure of the Generative Adversarial Networks is shown in Figure 1.

As shown in Figure 1, GAN usually uses random noise Z as the network input to generate the sample X_fake_ through the Generator network, and the Discriminator network is responsible for determining whether the input sample is the real sample X_real_ or the generated sample X_fake_. The objective function of the network training is as follows.

(1) $$\underset{G}{\min}\;\underset{D}{\max}V(D,G)=E_{x\sim p_{r}}[\log D(x)]+E_{s\sim p_{s}}[\log(1-D(G(z)))]$$

where *P_r_* denotes the true data distribution, *P_s_* denotes the generated data distribution, and *E* denotes the expectation.

During the training of the model, the discriminator *D* suffers from a two-category problem. *D* needs to be as accurate as possible in determining whether the input information is true data 1 or generated data 0. Its objective is to maximize the value of logD(x) and minimize the value of D(G(Z)). The objective function is the cross-entropy loss function V(D,G), which is common in two-category problems. For generator *G*, if it has a fixed discriminator *D*, it is necessary to cause the discriminator to determine generated data 0 as true data 1. An alternative interpretation is to maximize the discriminatory probability D(G(Z)) of the generated sample while minimizing log(1−D(G(Z))).

To improve the discriminator effect, we cause the generator and the discriminator to be trained alternately, keeping the parameters of the generator *G* unchanged when training the discriminator *D*. The parameters of the generator *G* are also kept unchanged. To improve the ability of generating data, it is ensured that the discriminator *D* parameters keep up to date during the training process while the generator *G* is being trained. Training is repeated over and over again until the performance of both networks is optimized. Usually, for the stability of the model training, the discriminator *D* is trained k times and the generator is iterated once more to ensure that the discriminator wins with a certain margin, usually set at *k* = 3. Finally, when the training is completed, the optimal discriminator *x* can be obtained by fixing the generator *G* and deriving the objective function.

Currently, the main improvement direction of Generative Adversarial Networks is to optimize the structure and performance of the model based on standard Generative Adversarial Networks to improve the quality of the generated images. Existing applications are mainly concentrated in the fields of image synthesis, super-resolution reconstruction, natural language processing, etc. Besides, good results have been achieved in some non-traditional application areas, such as medical data enhancement, image repair, and style migration. Generative Adversarial Networks (GAN) is a type of unsupervised learning, which does not rely on any a priori assumptions, and in terms of the universality of neural network applications, data generation can be done wherever there are data. Furthermore, since the sample generation is very simple, Generative Adversarial Networks are easy to port and build, and have a wider range of applications compared to traditional data generation methods.

### 3.2. Improved Generative Adversarial Networks

The main reason for the instability of GAN is that the model training is too free and difficult to converge. The training process is prone to the problem of model collapse, and the Nash equilibrium point cannot be reached after falling into a local optimum [29,30]. To address this problem, many solutions are proposed in the derivative model of GAN. Speeding up the convergence of the model by adding effective regular term constraints, such as Deep Infomax (DIM), was proposed by Hjelm et al. [31]. Due to the generalizability of neural networks, network structures with high performance can all provide some reference when facing different data generation tasks.

**Figure 2:**
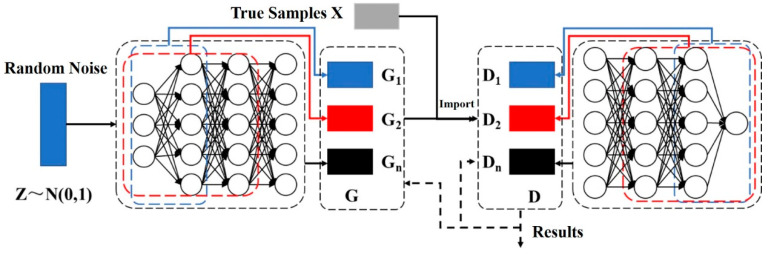
The framework of the improved Generative Adversarial Networks framework.

In this paper, when combining the problem of traffic accident data generation, the improved GAN is applied to generate some important features to achieve the purpose of increasing the strength of the raw traffic data. To this end, combined with many improvements of existing GAN, this paper proposes a traffic accident data generation model based on GAN, with the neural network structure shown in Figure 2.

The task of the generator is more difficult than that of the discriminator because of the work required to fit the probability density of the data. The discriminator only needs to discriminate among input samples, and it is this mechanism that leads to poor generator performance and a tendency for GAN to generate highly similar samples. Our approach of using a single discriminator with multiple generators is an effective way to mitigate this problem. Inspired by the structure of the DenseNet [32] convolutional neural network model, we will generate the adversarial neural network using multiple generators with multi-channel connections to form the final generative network.

As shown in Figure 2, the model is mainly divided into two parts, generator *G* and discriminator *D*. The generator consists of a fully connected artificial neural network, which is divided, according to the number of network layers, into multiple generators such as *G_1_*, *G_2_*, and *G_n_*. In addition, the connection is achieved by establishing shortcut channels between the network layers to form the final generator model. The underlying neurons of the generators share parameter weights and are input into the network by Gaussian distributed random noise to generate virtual samples. The discriminator consists of fully connected neural networks with a different number of layers to form different discriminators *D_1_*, *D_2_*, and *D_n_*, and one of them is selected as the discriminator of the network. Among them, the output of the generator and the input of the discriminator are structurally consistent, both being the length of the real sample *X*. The output of the generator and the input of the discriminator are identical in structure. The pseudocode of the generation process is shown in Algorithm 1.
**Algorithm 1.** Traffic Data Generation The stochastic gradient descent training process of the improved GAN. **Parameter *k*:** The ratio of the frequency of updating the generator to updating the discriminator. **Parameter *j*:** The upper limit of the number of iterations. **Begin**1. **for** *j* = 1…*j*2.   **for** *k* = 1…*k*3.   Calculate m samples *G*_1_(*Z*_1_, *Z*_2_...*Z_m_*), G_2_(*Z*_1_, *Z*_2_...*Z_m_*), *G_n_*(*Z*_1_, *Z*_2_...*Z_m_*) generated by Z through different generators, mix them to form the final generated sample *G*(*Z*_1_, *Z*_2_...*Z_m_*)4.   Extract m real samples *X* (*x*_1_, *x*_2_... *x_m_*)5.   Updated discriminator parameters: $D\leftarrow \nabla_{\theta_{d}}\frac{1}{m}\sum_{i=1}^{m}\left[\log D(x_{m})+\log(1-D(G(Z_{m})))\right]$6.   **end for**7.  Extract m generating samples separately *G*_1_(*Z*_1_, *Z*_2_…*Z_m_*), *G*_2_(*Z*_1_, *Z*_2_…*Z_m_*), *G_n_*(*Z*_1_, *Z*_2_…*Z_m_*)8.   Update generator parameters: $G\leftarrow \nabla_{\theta_{g}}\frac{1}{m}\sum_{i=1}^{m}\log(1-D(G(Z^{\left(i\right)})))$9. **end for**

## 4. Experimental Setup

### 4.1. Parameter Settings for Traffic Accident Data Generation

The improved GAN proposed in this study uses multiple generators in the form of full connections to generate data. This is done by establishing fast connections between the different neural network layers and enhancing the transfer of features between the different layers to optimize the training speed of the model. As shown in Figure 2, the generator and discriminator use different layers of fully connected neural networks for data generation and discrimination, respectively, and the specific neuronal parameters of each network are shown in Table 1. To prevent overfitting of the model and to reduce the number of parameters of the model, the various generators share the underlying neuronal parameters. Among them, while the first layer of the generator neural network uses the sigmoid activation function [33], all other layers use the ReLU activation function [34] to avoid the gradient disappearance phenomenon in the process of network training.

**Figure 3:**
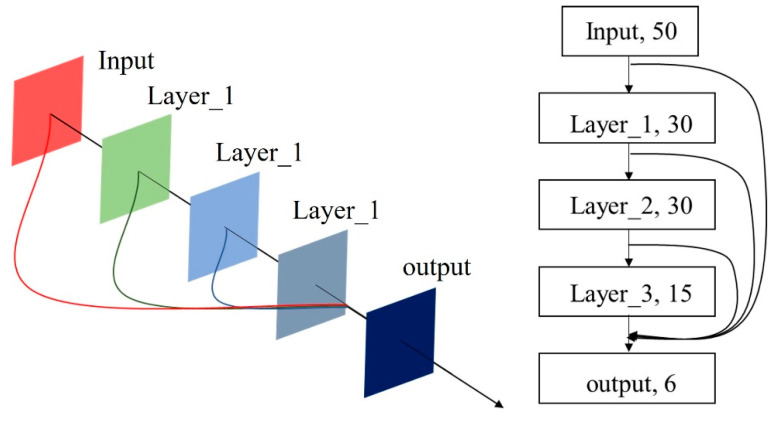
Schematic diagram of the generator structure.

The specific structure of the generator is shown in Figure 3, and the input from the output layer is derived from the output results of all previous network layers. The generator network contains a total of 131 neurons, and the network shares the underlying neuron parameters. The reason for using Sigmoid in the generator input layer is that Sigmoid works very well when there is a significant difference in features, which is continuously enhanced as the cycle progresses. However, the biggest disadvantage of the ReLU function, although it converges quickly, is that the gradient is 0 when the input is less than zero, which results in the negative gradient being set to 0 and the neuron being inactivated. It does not respond to any data, especially when the learning rate is large, which is often small with large neuronal necrosis generators, so ReLU can be used in the remaining layers to speed up training.

**Figure 4:**
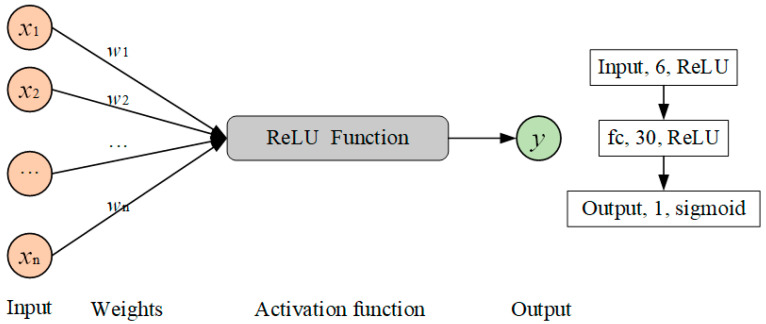
Schematic diagram of the discriminator structure.

The discriminator consists of a fully connected network containing one hidden layer. Since the discriminator deals with simple two-category problems, the model only sets a small number of neurons to avoid overfitting the model. The specific parameters are shown in Figure 4, except that the output layer uses the sigmoid activation function, and the other two layers use the Relu activation function.

Throughout the training process of generating the adversarial neural network, the input of the generator is noisy data of 50 dimensions and the output is a feature vector with a length of 6; the input of the discriminator is divided into two kinds, one is the feature vector from the original data and the other is the feature vector generated by the generator, and the output is the two-category result.

### 4.2. Typical Classifiers Used in the Experiments

For the fairness of the experiment, the following classification models are selected to compare the results of the experiments using the base model, the model without adding additional improvement measures, and the improved GAN in this paper. The four models are also used for the recognition of traffic accidents, and their recognition results are used to check whether the generated data are close to the original data.

1. Convolutional Neural Network (CNN)

The CNN is implemented based on the TensorFlow environment, and the network structure refers to the Convolutional Neural Network (CNN) part of the hybrid neural network. The model is solved by Adma, an optimization algorithm with random gradient descent, and the maximum number of iterations is set to 10,000. A certain amount of pre-processing is needed before the experiment, and the six-feature data are transformed into graph structure data after standardization operation.

2. Support Vector Machine (SVM)

The SVM belongs to a kind of two-category algorithm, which can map the features from the low dimension to the high dimension and construct a suitable hyperplane, so that the data that are linearly indistinguishable in the low-dimensional space can be linearly classified in the high dimension. The determination of the SVM hyperplane is determined by a few support vectors, which are less affected by redundant features, causing the algorithm to have good robustness. The accuracy of model classification is greatly influenced by the type of kernel function and related parameters, and the SVM focuses on the two categories, while multi-classing can only be achieved by indirect means.

3. K-Nearest Neighbor (KNN)

The KNN can be used for both linear and nonlinear classification, which is insensitive to outliers and has high classification accuracy. Its disadvantage is that it is not suitable for dealing with sample imbalance problems and cannot give the intrinsic meaning of the data.

4. Ridge Regression (RR)

Ridge regression is a biased estimation regression method dedicated to collinearity data analysis. It is essentially an improved least-squares estimation method. By abandoning the unbiasedness of the least square method, at the cost of loss of partial information and reduced accuracy, obtaining regression coefficients is more in line with the actual and more reliable regression method, and the fitting of ill-conditioned data is stronger than the least-square method. The model [35] assumes that there is a linear correlation between sample categories and characteristics that can satisfy a multivariate one-time equation. A loss function is constructed to solve for the parameters w and b, which are the parameters that minimize the distance between the predicted value and the classification value. The ridge regression model has a simple structure with good explanatory power but performs poorly when faced with non-linear classification problems.

To ensure experimental fairness, the SVM, KNN, and ridge regression all directly use the basic algorithm encapsulated by the sklearn library. The SVM uses the linear kernel function, the KNN is set at *k* = 5, and the rest of the algorithm uses the default parameters.

According to the confusion matrix shown in Table 2, this experiment can verify data generation by calculating the accuracy of the prediction results, adopting the accuracy A and False Positive Rate (FPR) as the evaluation indexes, and the evaluation method is expressed as follows.

(2) $$A=\frac{\mathrm{TP}+\mathrm{TN}}{\mathrm{TP}+\mathrm{FP}+\mathrm{FN}+\mathrm{TN}}$$

(3) $$\mathrm{FPR}=\frac{\mathrm{FP}}{\mathrm{FP}+\mathrm{TN}}$$

### 4.3. Description of the Traffic Accident Dataset

The raw dataset used in the experiments mainly consists of vehicle GPS data including the vehicle’s GPS positioning information, vehicle’s speed information, and vehicle’s position information, etc., collected from urban beltways and highways in Wuhan, Hubei, China, from 4 June to 10 June in 2018. After sample preprocessing and feature extraction (processing of 1000 GPS track information in five traffic accident scenarios), we finally obtain six features, namely the average acceleration, the standard deviation of acceleration, the standard deviation of speed, the average speed, the variance of acceleration, and the variance of speed, as shown in Table 3. The category labels are divided into two categories: Accident and normal traffic. Of these, 800 samples are used as training set samples and 200 samples are used as test set samples, and the data are normalized before it is fed into the classifiers.

To analyze the reliability of the generated samples, five quantitative indices are selected based on statistical principles, namely the sample average, maximum value, minimum value, median and sample variance, and the specific scores of each index are shown in Table 4. It consists of two parts, which are the scores of various statistical indicators for the original samples under normal traffic conditions and accident conditions, respectively.

## 5. Results and Discussion

### 5.1. Traffic Accident Data Generation

To keep the discriminator at a slight advantage during training, the update frequency ratio between the discriminator and the generator is *k* = 3. To prevent the problem of gradient disappearance during training, each layer of the generator and the discriminator uses batch normalization to batch normalize the input of the implicit layer. The experiment is solved by using the Adam optimizer with the learning rate size set to 0.0002, momentum to 0.5, and 64 samples processed per batch.

Table 5 consists of four sections, scoring the various statistical indicators for the original and generated samples in normal traffic and accident conditions, respectively. When analyzing the average speed characteristics of the vehicles, it can be seen that the generated data and the original data in the two states maintain similar levels, with the difference between the mean value not exceeding 1.3, the difference between the maximum and minimum value not exceeding 5, and the difference between the median value less than 4. When the variance values are similar but different, the accident data show the expected differences, indicating that the model is a good fit to the average speed of the vehicles. The same analysis shows that the data generation of average acceleration, acceleration standard deviation, and speed standard deviation is good, while the accident data generation of speed variance and acceleration variance features is average. Some of the value differences are too large. In the velocity variance feature, the difference between the variance values of the original samples and the generated sample is close to 90,000. The median and minimum values are also in different orders of magnitude, with there being a large difference between the maximum values.

To analyze whether there is a significant difference between the generated data and the original data, this paper firstly uses the F-test for two-sample ANOVA, secondly determines whether the variance is univariate according to the F-test results, and then performs the *t*-test, which mainly includes the two-sample equal variance hypothesis and the two-sample heteroskedasticity hypothesis. The specific test results are shown in Table 6.

From Table 6, it can be seen that the *p*-value of the F-test results of all generated data is greater than 0.05, illustrating that the variance between generated data and original data is homogeneous, and the *t*-test selects the two-sample equal variance hypothesis. It is worth noting that the *t*-test result of acceleration variance is 0.0581, close to 0.05. It indicates that, under normal conditions, the effect of acceleration variance of generating data is poor. Overall, there is no significant difference between the generated data and the original data, with the average velocity feature and the average acceleration feature generating better results.

### 5.2. Comparison Results of Accuracy of Traffic Accident Recognition

**Figure 5:**
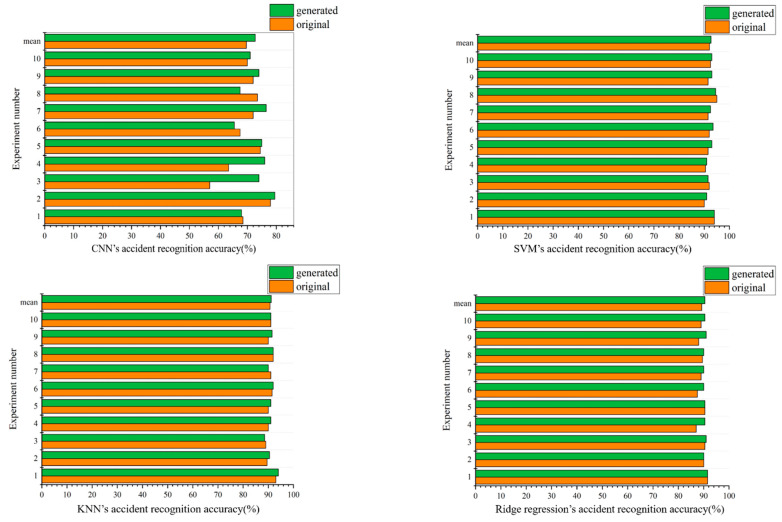
Comparison of accident recognition accuracy of each classifier trained on different datasets. The “generated” in the figure represents the “original + generated” data.

To further check the quality of the generated data, classification experiments are conducted for the recognition of traffic accidents in both the original and original + generated data. CNN, SVM, KNN, and ridge regression encapsulated by the sklearn library in Python are applied to traffic accident recognition. SVM uses the linear kernel function, with KNN being set at *k* = 5 and the rest of the algorithms using the default parameter settings. After ten-fold cross-validation, the average recognition results are obtained (shown in Figure 5). The value of experiment number 11 is the average value of the previous 10 experiments. By comparing the accuracy rate, it is found that when only the original dataset is used, the best performance of the classifier is SVM, with an average recognition rate of 92.05%. The KNN’s accident recognition rate is 90.7%, the ridge regression’s accident recognition rate is 89.25%, and the CNN’s accident recognition rate is 69.65%. When using the model trained on the original + generated dataset, the SVM’s accident recognition rate is 92.7%, the KNN’s accident recognition rate is 91.15%, the ridge regression’s accident recognition rate is 90.5% and the CNN’s accident recognition rate is 72.7%. Additionally, the accident recognition rate of the original + generated data increases up to 3.05%, which is higher than that of the original data. It can be seen that the quality of the generated data is very high.

### 5.3. Comparison Results of Traffic Accident Recognition

**Figure 6:**
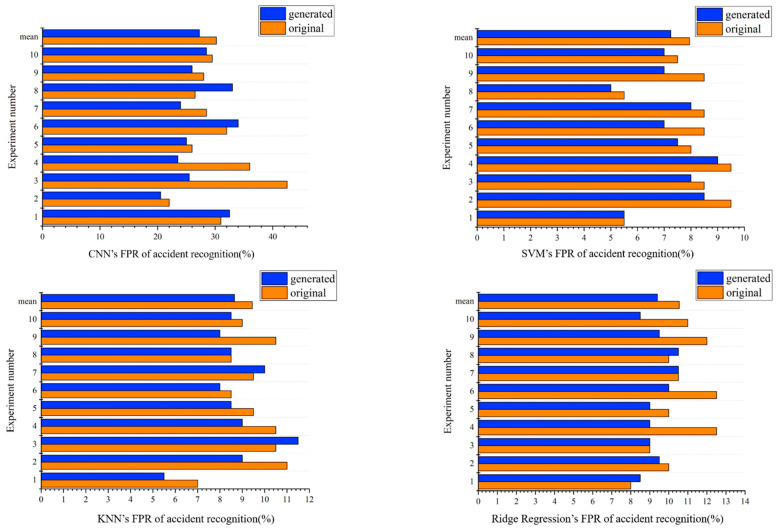
Comparison of FPR of accident recognition across classifiers trained on different datasets. The “generated” in the figure represents the “original + generated” data.

For the problem of traffic accident recognition, the FPR is an indicator of concern. The FPR refers to the probability that the recognition model judges a normal traffic event like a traffic accident. An excessive FPR will lower the reliability of the recognition model and using false alarm results will cause unnecessary losses. Each model uses different dataset FPR results as shown in Figure 6. The value of experiment number 11 is the average value of the previous 10 experiments. By comparing the FPR of accident recognition, it is found that when using only the original dataset, the FPR of accident recognition by SVM is 7.95%, the FPR of accident recognition by KNN is 9.45%, the FPR of accident recognition by ridge regression is 10.55%, and the FPR of accident recognition by CNN is 30.2%. When using the model trained on the original + generated dataset, accident recognition by SVM is 7.25%, the FPR of accident recognition by KNN is 8.65%, the FPR of accident recognition by ridge regression is 9.4%, and the FPR of accident recognition by CNN is 27.25%. Furthermore, the accident recognition FPR of the original + generated data reduces up to 2.95% compared with the accident recognition FPR of the original data. It can also be seen that the quality of the generated data is very high.

## 6. Conclusions

Traffic accidents are considered a major threat to safety due to their destructive power as low-probability emergencies. Identifying and responding to traffic accidents quickly and accurately and reducing the safety risks associated with them is a constant focus of experts in the field of transport. However, due to the small number of accident samples, academics are rarely able to analyze the specific characteristics of traffic accidents directly from the data. They often need to combine information from a variety of sources, such as road congestion status, manual feedback, traffic flow information, and so on. With the continuous development of technology, the information age provides more data channels, and data generation technology is also maturing. By generating the powerful data-generating capabilities of adversarial neural networks, we can model large amounts of traffic accident data to mine the deeper features of traffic accidents after obtaining sufficient data and providing new ideas for solving the traffic accident recognition problem.

The highlights of this paper are as follows: (1) Designing an improved GAN-based traffic accident data generation approach with a novel network structure. (2) To further check the quality of the generated data, extensive experiments are conducted and analyzed. It is concluded that the generated samples for each feature are not significantly different from the original data within the 95% confidence interval, and all *p*-values are greater than 0.05. Results of the traffic accident recognition also indicate that the augmented (original + generated) data significantly improve the accident recognition ability of the four selected classifiers. Specifically, the accuracy of accident recognition is increased by 3.05% at the maximum, and the FPR of accident recognition is reduced by 2.95% at the maximum, further supporting the reliability and validity of the proposed approach in practical applications.

This paper also has some limitations. First, the data collected from the urban beltways and highways only contain vehicle-related accidents, while those involving pedestrian and non-motor vehicle such as bikes or e-bikes are neglected in this study. Second, there is still room for improvement in the generative and discriminative models. Further research may include optimizing the structure of the generative adversarial neural network model to make the training process and the generative performance more efficient. Moreover, we will extend the application scenarios by including non-motor vehicle or pedestrian-related traffic accidents.

## Figures and Tables

**Table 1:** Global network parameters of the generators.

G_1_	G_2_	G_3_
Input fc, 50, sigmoid	Input fc, 50, sigmoid	Input fc, 50, sigmoid
Fc, 30, Relu	fc, 30, Relu	fc, 30, Relu
	fc, 30, Relu	fc, 30, Relu
		fc, 15, Relu
Concat		
fc, 6, Relu		

**Table 2:** Confusion matrix.

	Prediction Category	Accident	Normal
True Category			
Accident		True Positives (TP)	False Negatives (FN)
Normal		False Positives (FP)	True Negatives (TN)

**Table 3:** Description of the traffic accident dataset used in the experiments.

Feature Name	Abbreviation	Definition	Instruction
Average of Speed (km/h)	V_a_	$\bar{V}=\frac{1}{n}\sum \left(v_{1}+v_{2}+\ldots +v_{n}\right)$	Average of instantaneous speeds of vehicles over some time.
Variance of Speed (km^2^/h^2^)	Var_V	$S_{v}^{2}=\frac{1}{n}\sum_{i=1}^{n}{\left(v_{i}-\bar{V}\right)}^{2}$	The range of instantaneous speed value of the model in an instant.
Standard deviation of Speed (km/h)	Std_V	$\sigma (v)=\sqrt{\frac{1}{n}\sum_{i=1}^{n}{\left(v_{i}-\bar{V}\right)}^{2}}$	The standard deviation of the instantaneous speed value of the vehicle over some time.
Average of Acceleration (m/s^2^)	a_a_	$\bar{a}=\frac{1}{n}\sum \left(a_{1}+a_{2}+\ldots +a_{n}\right)$	The average value of the instantaneous acceleration value of the vehicle over some time.
Variance of Acceleration (m^2^/s^4^)	Var_a	$S_{a}^{2}=\frac{1}{n}\sum_{i=1}^{n}{\left(a_{i}-\bar{a}\right)}^{2}$	The variance of the instantaneous acceleration value of the vehicle over some time.
Standard deviation of Acceleration (m/s^2^)	Std_a	$\sigma (a)=\sqrt{\frac{1}{n}\sum_{i=1}^{n}{\left(a_{i}-\bar{a}\right)}^{2}}$	The standard deviation of the instantaneous acceleration value of the vehicle over some time.

**Table 4:** Description of the original samples under various statistical indicators.

Features		Average	Maximum	Minimum	Median	Variance
Average of speed (km/h)	normal	87.39	101.64	68.69	88.45	75.49
	accident	69.97	105.4	43.5	68.29	188.63
Average of acceleration (m/s^2^)	normal	0	0.13	−0.13	0.007	0.004
	accident	−0.19	−0.011	−0.394	−0.19	0.006
standard deviation of Speed (km/h)	normal	7.52	18.87	1.76	7.16	12.77
	accident	29.79	47.2	3.65	30.25	88.26
Standard deviation of acceleration (m/s^2^)	normal	0.5	1.02	0.12	0.58	0.050
	accident	1.18	1.91	0.72	1.11	0.12
Variance of velocity (km^2^/h^2^)	normal	69.10	355.96	3.09	51.19	4269.10
	accident	973.96	2227.84	13.35	915.37	261,835.13
variance of Acceleration (m^2^/s^4^)	normal	0.35	1.04	0.01	0.33	0.061
	accident	1.50	3.65	0.52	1.22	0.82

**Table 5:** Description of original and generated samples under various statistical indicators.

Features			Average	Maximum	Minimum	Median	Variance
Average of speed (km/h)	normal	original	87.39	101.64	68.69	88.45	75.49
		generated	86.99	102.18	66.26	87.24	54.29
	accident	original	69.97	105.4	43.5	68.29	188.63
		generated	71.24	100.8	48.05	71.90	142.01
Average of acceleration (m/s^2^)	normal	original	0	0.13	−0.13	0.007	0.004
		generated	0.006	0.14	−0.11	0.010	0.003
	accident	original	−0.19	−0.011	−0.394	−0.19	0.006
		generated	−0.18	−0.01	−0.323	−0.17	0.005
standard deviation of speed (km/h)	normal	original	7.52	18.87	1.76	7.16	12.77
		generated	8.74	17.21	1.06	8.82	15.94
	accident	original	29.79	47.2	3.65	30.25	88.26
		generated	30.88	47.12	13.23	30.77	49.66
Standard deviation of acceleration (m/s^2^)	normal	original	0.5	1.02	0.12	0.58	0.050
		generated	0.656	1.06	0.15	0.68	0.039
	accident	original	1.18	1.91	0.72	1.11	0.12
		generated	1.13	1.88	0.59	1.08	0.09
Variance of velocity (km^2^/h^2^)	normal	original	69.10	355.96	3.09	51.19	4269.10
		generated	80.96	232.34	3.80	68.89	2541.35
	accident	original	973.96	2227.84	13.35	915.37	261,835.13
		generated	1127.51	2702.43	175.11	1067.94	355,275.85
Variance of Acceleration (m^2^/s^4^)	normal	original	0.35	1.04	0.01	0.33	0.061
		generated	0.44	1.07	0.02	0.40	0.059
	accident	original	1.50	3.65	0.52	1.22	0.82
		generated	1.31	2.84	0.35	1.16	0.43

**Table 6:** Test for significant differences between generated and original samples.

Features		*p*-Val (F-Test)	*p*-Val (*t*-Test)	Significant Differences
Average of speed (km/h)	normal	0.1260	0.8032	NO
	accident	0.1618	0.6250	NO
Average of acceleration (m/s^2^)	normal	0.1268	0.5479	NO
	accident	0.3434	0.7360	NO
Standard deviation of speed (km/h)	normal	0.2201	0.1099	NO
	accident	0.1233	0.5139	NO
Standard deviation of acceleration (m/s^2^)	normal	0.2266	0.1138	NO
	accident	0.1748	0.4630	NO
Variance of velocity (km^2^/h^2^)	normal	0.1362	0.3120	NO
	accident	0.1444	0.1700	NO
Variance of Acceleration (m^2^/s^4^)	normal	0.4760	0.0581	NO
	accident	0.1136	0.2244	NO

## Data Availability

Not applicable.
